# *Chayamaritia vietnamensis* (Gesneriaceae), a new species from Son La Province, northern Vietnam

**DOI:** 10.3897/phytokeys.177.63401

**Published:** 2021-05-12

**Authors:** Zi-Bing Xin, Long-Fei Fu, Stephen Maciejewski, Zhang-Jie Huang, Truong Van Do, Fang Wen

**Affiliations:** 1 Guangxi Key Laboratory of Plant Conservation and Restoration Ecology in Karst Terrain, Guangxi Institute of Botany, Guangxi Zhuang Autonomous Region and Chinese Academy of Sciences, CN-541006, Guilin, China Gesneriad Conservation Center of China Guilin China; 2 Gesneriad Conservation Center of China (GCCC), National Gesneriaceae Germplasm Bank of GXIB, Guilin Botanical Garden, Guangxi Zhuang Autonomous Region and Chinese Academy of Sciences, CN-541006 Guilin, China Guangxi Institute of Botany Guilin China; 3 The Gesneriad Society, 2030 Fitzwater Street, Philadelphia, PA, 19146-1333, USA Gesneriad Committee, China Wild Plant Conservation Association Guilin China; 4 Vietnam National Museum of Nature, Vietnam Academy of Science & Technology, 18 Hoang Quoc Viet, Hanoi, Vietnam The Gesneriad Society Philadelphia United States of America; 5 Graduate University of Science and Technology, Vietnam Academy of Science & Technology, 18 Hoang Quoc Viet, Hanoi, Vietnam Vietnam National Museum of Nature, Vietnam Academy of Science &amp; Technology Hanoi Vietnam; 6 Gesneriad Committee, China Wild Plant Conservation Association, CN-541006, Guilin, China Graduate University of Science and Technology Hanoi Vietnam

**Keywords:** Cliff-dwelling, Flora of Vietnam, new taxon, taxonomy

## Abstract

*Chayamaritia vietnamensis*, a new species from Son La Province, northern Vietnam, is described and illustrated. The phylogenetic study revealed that the new species is most closely related to *C. banksiae* and *C. smitinandii*. The morphological comparison suggests it as the third new species of *Chayamaritia* and distinguished from *C. banksiae* and *C. smitinandii* by a combination of morphological characters of leaf blades, bracts, calyx and corolla, especially its peltate leaf blades. This species is provisionally assessed as endangered (EN B1ab(iii), B2ab(iii)) using IUCN Categories and Criteria. Information on ecology, phenology and an identification key for the known *Chayamaritia* species are also provided.

## Introduction

The genus *Chayamaritia* D.J.Middleton & Mich.Möller (Gesneriaceae) was originally established and described, based on molecular and morphological data (Middleton et al. 2015). The genus *Chayamaritia* comprises two species and is hitherto known only in Laos and Thailand. A thickened rhizomatous prostrate stem, along with alternately arranged leaves and the imbricate sepals characterise the genus (Middleton et al. 2015). The type species, *Chayamaritia smitinandii* (B.L.Burtt) D.J.Middleton, was initially placed in the genus *Chirita* Buch.-Ham. (Burtt, 2001), then transferred to the genus *Henckelia* Spreng. (Weber et al. 2011) and eventually separated as a new genus in 2015, together with the newly-described species, *Chayamaritia banksiae* D.J.Middleton (Middleton et al. 2015). *Chayamaritia banksiae* and *C. smitinandii* are endemic to Laos and Thailand, respectively (Middleton et al. 2015).

During a joint Sino-Vietnamese botanical survey for plant biodiversity in northern Vietnam in late October 2019, we collected an unknown Gesneriaceae plant. In the Xuan Nha Nature Reserve, Moc Chau District, Son La Province, northern Vietnam, we discovered this plant that looked like a *Chayamaritia* species. Detailed comparison of these specimens with the type specimens and protologues of known *Chayamaritia* species showed that these specimens neither fitted the existing protologues nor conformed to the type specimens of these species. Although, the leaf indumentum and inflorescence of the unknown plant was most similar to those of *C. banksiae* and *C. smitinandii*, it could be easily distinguished from the latter two by the combination of several morphological characters of the leaf blades, bracts, calyx and corolla, especially its peltate leaf blade. Thus, we confirmed that it represented a new species of *Chayamaritia*, which is the first *Chayamaritia* species from Vietnam. The description, illustration, information on ecology, phenology and provisional conservation assessment using IUCN (2019) of the proposed new species are provided. Furthermore, an identification key to the known *Chayamaritia* species is given.

## Material and methods

### Plant material

Herbarium materials were studied from the following herbaria: E, IBK, US and VNMN (herbarium acronyms according to Index Herbariorum; Thiers 2019). The macromorphological features were observed, based on the specimen sheets and notes in both the field and the conservation nursery at the Gesneriad Conservation Center of China and the National Gesneriaceae Germplasm Bank at GXIB. Micromorphological features were analysed and photographed using an optical microscope (Stemi DV4, LEICA S8 AP0, Jena, Germany).

These morphological characters of newly-proposed species were compared with those of the two known *Chayamaritia* species from protologues, type specimens and living plants. The description of the new species followed the terminology used by Harris and Harris (2001) and Wang et al. (1998). Assessment of the conservation status of the new species was according to Categories and Criteria of the IUCN (2019).

### DNA extraction, PCR amplification and sequencing

Leaves were dried using silica gel for DNA extraction (Chase and Hills 1991). Genomic DNA was extracted using the CTAB protocol (Doyle and Doyle 1987). To confirm the placement of this new species, we performed phylogenetic studies of DNA sequence data obtained from the nuclear ribosomal internal transcribed spacer (ITS) region and the plastid *trnL-F* intron spacer (*trnL-F*). Given the phylogenetic studies of Middleton et al. (2015), we sampled two species (three accessions) from *Chayamaritia* and the new species as ingroup and ten species from all closely related and morphologically similar genera, including *Allostigma* (one species), *Deinostigma* (two species), *Loxostigma* (two species), *Petrocosmea* (two species), *Pseudochirita* (two varieties) and *Primulina* (two species) as outgroup (Middleton et al. 2015). DNA extraction, PCR amplification and sequencing were performed following Wei et al. (2013). The species name, voucher specimens and GenBank accession numbers of DNA sequences used in this study are listed in Table 1.

**Table 1.** Species names, voucher specimens, and GenBank accession numbers of DNA sequences used in this study.

Species name	Voucher number	Herbarium	Origin	ITS	*trnL-F*
*Allostigma guangxiense* W.T.Wang	M. Möller and Y.G. Wei MMO 05-755	E, IBK	China, Guangxi, Longzhou county	HQ632977	HQ632880
*Deinostigma cicatricosa* (W.T.Wang) D.J.Middleton & Mich.Möller	W. B. Xu s.n. [XWB]	IBK	China, unknown locality	JX506925	JX506817
*Deinostigma cyrtocarpa* (D.Fang & L.Zeng) Mich.Möller & H.J.Atkins	M. Möller and Y.G. Wei MMO 06-908	E, IBK	China, Guangxi, He Zhou city	KU990889	KU990885
*Pseudochirita guangxiensis* (S.Z.Huang) W.T.Wang	M. Möller and Y.G. Wei MMO 06-798	E, IBK	China, Guangxi, Mashan county	HQ633003	HQ632908
Pseudochirita guangxiensis (S.Z.Huang) W.T.Wang var. glauca Y.G.Wei & Yan Liu	M. Möller and Y.G. Wei MMO 05-751	E, IBK	China, Guangxi, Jingxi county	HQ633004	HQ632909
*Loxostigma glabrifolium* D.Fang & K.Y.Pan	Y.G. Wei 709	IBK	China, Guangxi, Napo county	HQ633006	HQ632910
*Loxostigma griffithii* (Wight) C.B.Clarke	Kew/Edinburgh Kanchenjunga Expedition (1989) 940 [Cult. RBGE 19892473A]	E	Nepal, Yamphudin	FJ501338	FJ501508
*Chayamaritia banksiae* D.J.Middleton	D.J. Middleton 5220 and M. Newman et al. LAO1428	E	Laos, Khammouan, Nakai Nam Theun	KP325426	KP325433
*Chayamaritia smitinandii* (B.L.Burtt) D.J.Middleton & Mich.Möller	D.J. Middleton et al. 5632	E	Thailand, Nakhon Nayok, Khao Yai NP	KP325424	KP325431
*Chayamaritia smitinandii* (B.L.Burtt) D.J.Middleton & Mich.Möller	D.J. Middleton et al. 5652	E	Thailand, Nakhon Nayok, Khao Yai NP	KP325425	KP325432
*Chayamaritia vietnamensis* F.Wen, T.V.Do, Z.B.Xin & S.Maciej	F. Wen, T.V. Do, Z.B. Xin & S. Maciejewski, VMN-CN1214	IBK, VNMN	Vietnam, Son La, Moc Chau	MW458944*	MW458945*
*Petrocosmea kerrii* Craib	Voucher from Cult. RBGE 19715592	E	unknown origin	FJ501334	FJ501502
*Petrocosmea nervosa* Craib	Smithsonian Institute 78-057 [Cult. RBGE 19933232]	E, US	China, N Yunnan	FJ501335	AJ492299
*Primulina tabacum* Hance	Q.J. Xie and C.X. Ye s.n. [Cult. RBGE 19951540]	E	E China, Guangdong, Lian Rive	FJ501352	AJ492300
*Primulina gemella* (D.Wood) Yin Z.Wang	L. Averyanov 1987 [Cult. RBGE 19941913]	E	Vietnam, Hong Quang Special Region, Cat Hai	FJ501345	FJ501523

Note: newly generated sequences indicated by an asterisk (*).

### Phylogenetic analysis

The sequence data were edited and assembled using Lasergene Navigator 7.1 (DNAstar, Madison, Wisconsin, USA). Two datasets (ITS and *trnL-F*) were aligned independently using MAFFT version 7.0 (Katoh and Standley 2013) with default settings. The two best-supported tree topologies from Maximum Likelihood (ML) analyses of ITS and *trnL-F* were compared visually for topological incongruences. As there were no hard incongruences (Nishii et al. 2015), phylogenies were reconstructed, based on the combined dataset using ML and Bayesian Inference (BI). BI was performed using MRBAYES v.3.2.7 (Ronquist et al. 2012). Best-fitting models for the BI analysis were obtained independently for each gene region using MODELTEST v.3.7 (Posada and Buckley 2004). GTR+G and GTR+I were the best-fitting models for ITS and *trnL-F*, respectively. One cold and three incrementally heated Markov Chain Monte Carlo (MCMC) chains were run for five million generations and trees were sampled every 1,000 generations (5,000 trees sampled in total). The first 1250 trees (25%) were discarded as burn-in prior to calculating the BI consensus trees and posterior probabilities (PP) (See Suppl. material 1: log file). The ML analyses were performed in RAxML using raxmlGUI (Silvestro and Michalak 2012), with GTRGAMMA setting and 1,000 bootstrap replicates.

## Results

**Figure 1.**
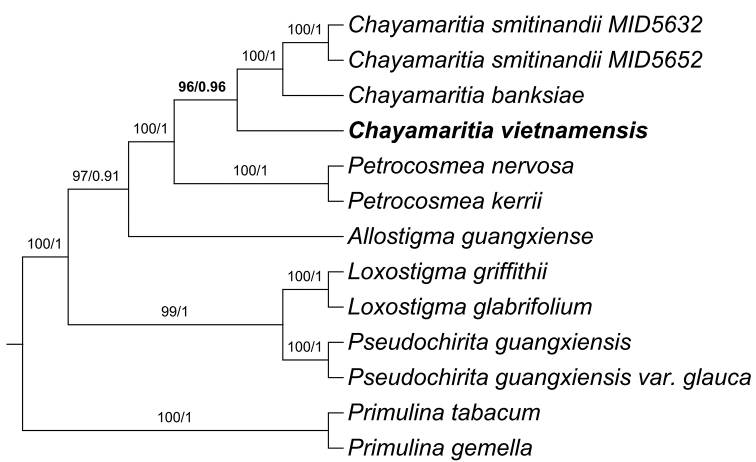
Phylogenetic tree of *Chayamaritia* and related genera generated from the Maximum Likelihood analysis (ML) of the combined dataset (ITS and *trnL-F*). Numbers on the branches indicate bootstrap values (≥ 50%) of the ML and the posterior probability (≥ 0.5) of the BI analyses. Created by Long-Fei Fu.

The combined ITS and *trnL-F* matrix was 1,477 characters long (700 for ITS and 777 for *trnL-F*). Of the 378 variable characters, 155 (56.97%) were parsimony-informative. ML and BI analyses resulted in the same tree topology indicating the undescribed species as sister to the two known *Chayamaritia* species (BS = 97%, PP = 1), i.e. *C. banksiae* and *C. smitinandii* (Fig. 1).

### Taxonomic treatment

#### Chayamaritia vietnamensis

Taxon classificationPlantaeLamialesGesneriaceae

F.Wen, T.V.Do, Z.B.Xin & S.Maciej sp. nov.

38547DF8-4D4C-5992-9EB2-2DB9DDB19E9D

urn:lsid:ipni.org:names:77217099-1

Figs 2 , 3C

##### Diagnosis.

The new species can be easily distinguished from the known *Chayamaritia* species by its peltate leaf blades. Besides, it differs from *C. banksiae* by its leaf blades apex rounded and margin entire (vs. apex shortly acuminate and margin minutely dentate); bracts 3, apex rounded and margin entire (vs. 2, apex acuminate and margin dentate); calyx lobes inside glabrous and margin entire (vs. inside with white appressed hairs in upper half, margin coarsely dentate); corolla lobes margin entire (vs. margin being minutely dentate); lateral staminodes 2.5–4 mm long (vs. 5.5–11 mm long). It also differs from *C. smitinandii* by its leaf blades apex rounded and margin entire (vs. apex acuminate and margin minutely dentate); bracts 3, ovate narrow and apex rounded (vs. 2, narrowly elliptic to lanceolate, somewhat falcate, apex acuminate); calyx lobes inside glabrous and margin entire (vs. inside densely pubescent, margin slightly toothed or appearing as large sessile glands on margin).

##### Type.

Vietnam. Son La Province: Moc Chau District, Xuan Nha Nature Reserve, 20°43'N, 104°40'E, elev. ca. 850 m, 31 October 2019, *F. Wen*, *T.V. Do*, *Z.B. Xin* & *S. Maciejewski*, *VMN-CN1214* (Holotype: VNMN!; Isotypes: IBK!, VNMN!).

##### Description.

Herbs perennial, rhizomatous prostrate thickened stem. *Leaves* basal, alternately arranged, numerous; *petioles* cylindrical, 10–25 cm long, 6–8 mm in diameter, densely covered with short white appressed hairs; *leaf blade* ovate to elliptic, peltate, 12–20 × 10–15 cm, 1.2–1.3 times as long as wide, both surfaces densely covered with short white appressed hairs, base rounded, apex rounded, margin entire; lateral veins 6–9 on each side of the mid-rib, impressed on the adaxial surface, prominent on the abaxial surface. *Inflorescences* cymose, all axes and bracts pale green with red appressed hairs; cymes 4–6, axillary, 1–3-branched, 2–12-flowered; *peduncle* 15–25 cm long, 4–6 mm in diameter, scattered villous; *bracts* 3, narrow ovate, 1.3–1.6 cm long, 5–6 mm wide, adaxially sparsely villous, abaxially densely villous, margin entire, apex rounded; *pedicel* 2.5–3.5 cm long, 1.5–2 mm in diameter, spreading puberulent. *Calyx* 5-parted nearly to the base, strongly imbricate; *lobes* ovate, ca. 1.3 cm long, ca. 6 mm wide, appressed villous outside, glabrous inside, margin entire, apex acuminate. *Corolla* 5.5–6.5 cm long, dark purple throughout outside, white to pale purple with two parallel yellow lines ventrally inside, lobes purple outside and inside, paler at base; *tube* 3.5–4 cm long, 1–1.2 cm in diameter at the mouth, 6–8 mm in diameter at the base; *limb* distinctly 2-lipped, *adaxial lip* 2-parted to over middle, lobes ca. 1 × 1 cm, orbicular; *abaxial lip* 3-parted to near the middle, lobes 1.3–1.5 × ca. 1 cm, oblong. *Stamens* 2, adnate to ca. 2.2 cm above the base of the corolla tube; *filaments* 1–1.2 cm long, white, sparsely pubescent, strongly geniculate at ca. 5 mm above the filament base, *anthers* ca. 2 mm long, sparsely pubescent. *Staminodes* 3, lateral ones 2.5–4 mm long, white, glabrous, adnate to 1.8 cm above the base of the corolla tube, the middle one ca. 0.5–1 mm long, adnate to 1.2 cm above the base of the corolla tube. *Disc* orbicular, ca. 3 mm in height, 5-crenate at the margin, glabrous. *Pistil* 4–4.5 cm long, *ovary* 2.5–2.8 cm long, 2–2.5 mm in diameter, mixed pubescent and glandular-pubescent; *style* 1–1.2 mm long, ca. 0.6 mm in diameter, mixed pubescent and glandular-pubescent; *stigma* only of lower lobe, bifid with blunt lobes, lobe ca. 3 mm long, ca. 0.5 mm in diameter. *Capsules* straight, 5.5–6.5 cm long, ca. 3.5–4 mm in diameter.

**Figure 2.**
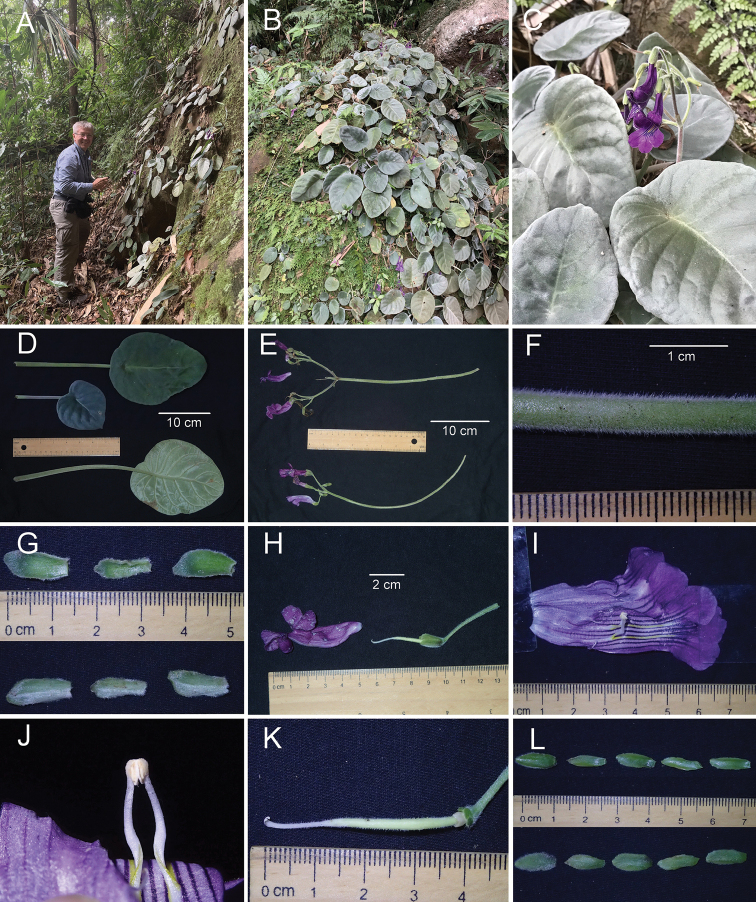
*Chayamaritia vietnamensis* F.Wen, T.V.Do, Z.B.Xin & S.Maciej **A, B** habitat **C** habit **D** adaxial (top) and abaxial (bottom) surface of leaf blade **E** cymes **F** peduncle **G** adaxial (top) and abaxial (bottom) surface of bracts **H** pistil, calyx and lateral view of corolla **I** opened corolla with stamens and staminodes **J** stamens with cohering anthers **K** pistil **L** adaxial (top) and abaxial (bottom) surface of calyx lobes. Photos by Fang Wen, arranged by Zi-Bing Xin.

##### Phenology.

Flowering occurs from October to December and fruiting from November to January.

##### Etymology.

The specific epithet “*vietnamensis*” is derived from Vietnam, which holds the first discovered and only known location for the species.

**Figure 3.**
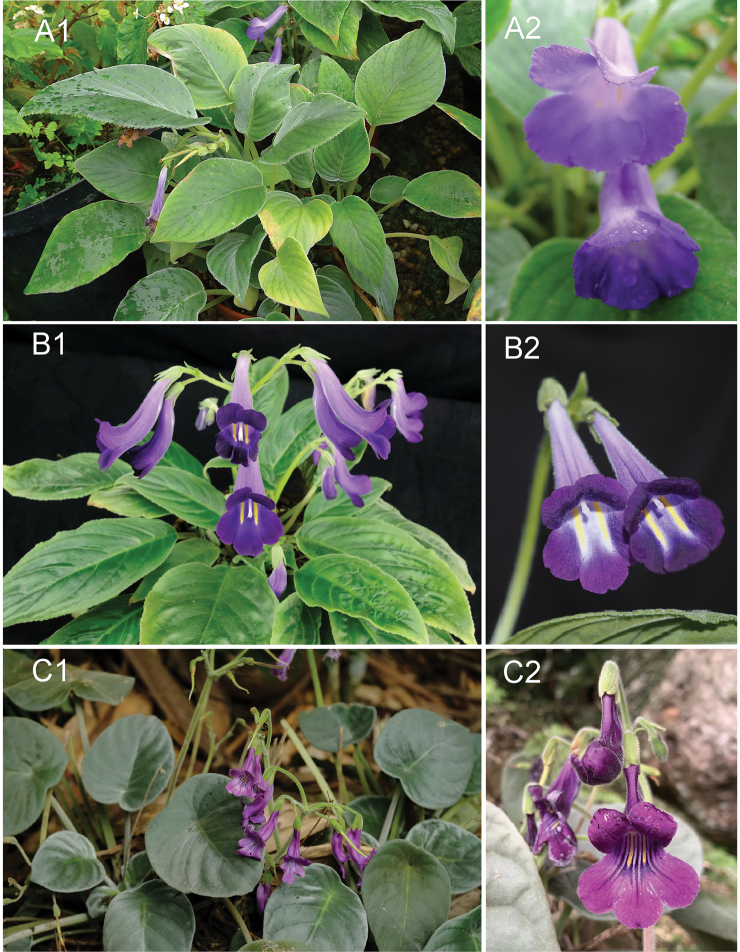
Three species of *Chayamaritia***A***C. banksiae* D.J.Middleton **B***C. smitinandii* (B.L.Burtt) D.J.Middleton **C***C. vietnamensis* F.Wen, T.V.Do, Z.B.Xin & S.Maciej. Photos by Fang Wen, arranged by Zi-Bing Xin.

##### Distribution and habitat.

*Chayamaritia vietnamensis* is hitherto only known from the type locality, Xuan Nha Nature Reserve, Moc Chau District, Son La Province, northern Vietnam. It grows on rock surfaces surrounded by limestone areas in a subtropical evergreen seasonal rain forest.

##### Conservation status.

*Chayamaritia vietnamensis* is known from two small-sized populations in the Xuan Nha Nature Reserve’s buffer zone. The EOO and AOO of the new species are about 6.15 km^2^ and 2.2 km^2^, respectively. Furthermore, the natural habitat is mostly disturbed by local farmers who impose intense pressure on the remaining primary forest patches, by converting the natural habitat of the species into cornfields. Thus, following the IUCN Red List Categories and Criteria (IUCN 2019), it is provisionally assessed as endangered (EN B1ab(iii), B2ab(iii))

### Identification key for the three known *Chayamaritia* species (Fig. 3)

**Table:** 

1	Leaf blade peltate, apex rounded, margin entire	***C. vietnamensis***
–	Leaf blade not peltate, apex acuminate, margin minutely dentate	**2**
2	Calyx lobes 7–15 mm wide, corolla tube white ventrally	***C. banksiae***
–	Calyx lobes 0.9–4.2 mm wide, corolla tube purple ventrally	***C. smitinandii***

## Discussion

From the viewpoint of morphology, the newly-described species should be treated as a species of *Chayamaritia*, because it exhibits a suite of morphological characters that are diagnostic to the genus and should not be established as a distinct genus. It has a thickened rhizomatous prostrate stem, alternately arranged leaves and imbricate sepals (Middleton et al. 2015). The current molecular work also confirmed that the new species is sister to a clade comprising the two known *Chayamaritia* species (*C. banksiae* and *C. smitinandii*). A detailed morphological comparison of the new species with its two relatives is summarised in Table 2. This new species is the first *Chayamaritia* species reported from Vietnam. In order to facilitate identification, we also provide an identification key to all known *Chayamaritia* species.

**Table 2.** Morphological comparison of *Chayamaritia vietnamensis* and its two relatives.

**Character**s	***C. vietnamensis***	***C. banksiae***	***C. smitinandii***
**Leaf blade**	peltate, 1.2–1.3 times as long as wide, apex rounded, margin entire	palaceous, 1.5–1.9 times as long as wide, apex shortly acuminate, margin minutely dentate	palaceous, 1.8–5.6 times as long as wide, apex acuminate, margin minutely dentate
**Bracts**	3, narrow ovate, 5–6 mm wide, apex rounded, margin entire	2, ovate, 19 mm wide, apex acuminate, margin dentate	2, narrowly elliptic to lanceolate, somewhat falcate, 1.8–8 mm wide, apex acuminate, margin entire
**Pedicels**	25–35 mm long	12–15 mm long	6.5–11 mm long
**Calyx lobes**	4–6 mm wide, inside glabrous, margin entire	7–15 mm wide, inside with white appressed hairs in the upper half, margin coarsely dentate	0.9–4.2 mm wide, inside densely pubescent, margin slightly toothed or appearing as large sessile glands on margin
**Corolla**	outside dark purple throughout, lobes margin entire	outside white ventrally, lobes being minutely dentate along the margin	outside deep purple throughout, lobes margin entire
**Lateral staminodes**	2.5–4 mm long	5.5–11 mm long	4–5 mm long
**Disc**	ca. 3 mm high	ca. 1.5 mm high	0.9–1.4 mm high

## Conclusion

In the present study, we discovered and described a new species of *Chayamaritia* from Vietnam, based on both morphological and molecular evidence. This newly-described species further suggests floristic similarities amongst countries of the Indochinese Peninsula. Our new finding provides an essential addition to the ongoing project of ‘Flora of Vietnam’ and ‘Flora of Cambodia, Laos and Vietnam’.

## Supplementary Material

XML Treatment for Chayamaritia vietnamensis

## Supplementary materials

Supplementary material 1 Log file Data type phylogenetic https://binary.pensoft.net/file/541613This dataset is made available under the Open Database License (http://opendatacommons.org/licenses/odbl/1.0/). The Open Database License (ODbL) is a license agreement intended to allow users to freely share, modify, and use this Dataset while maintaining this same freedom for others, provided that the original source and author(s) are credited.Zi-Bing Xin, Long-Fei Fu, Stephen Maciejewski, Zhang-Jie Huang, Truong Van Do, Fang Wen
